# Metabolomics combined with transcriptomics and physiology reveals the regulatory responses of soybean plants to drought stress

**DOI:** 10.3389/fgene.2024.1458656

**Published:** 2024-10-24

**Authors:** Liwei Wang, Peijin He, Mengmeng Hui, Hainan Li, Anni Sun, Hong Yin, Xining Gao

**Affiliations:** ^1^ College of Agronomy, Shenyang Agricultural University, Shenyang, China; ^2^ Liaoning Key Laboratory of Agrometeorological Disasters, Shenyang, China; ^3^ Liaoyang Meteorological Bureau, Liaoyang, Liaoning, China; ^4^ Anshan Meteorological Bureau, Anshan, Liaoning, China

**Keywords:** soybean, drought stress, metabolomics, transcriptomics, proline, spermidine

## Abstract

Drought, a prevalent environmental stressor, has had significant consequences on soybean (*Glycine max* L.), notably impeding its growth and production. Therefore, it is crucial to gain insight into the regulatory responses of soybean plants exposed to drought stress during soybean flowering in the field. In this study, the cultivar ‘Liaodou 15’ was performed light drought (LD, 24.3% soil moisture content), moderate drought (MD, 20.6% soil moisture content) and severe drought (SD, 16.9% soil moisture content) treatments at flowering stages of soybean and then rehydrated (30% soil moisture content) until harvest. The yield-related indicators were measured and revealed that MD and SD treatments significantly reduced 6.3% and 10.8% of the 100-grain weight. Soybean plants subjected to three drought stresses showed that net photosynthetic rates were 20.8%, 51.5% and 71.8% lower in LD, MD and SD than that of CK. The WUE increased by 31.8%, 31.5% and 18.8% under three drought stress treatments compared to CK. In addition, proline content was 25.94%, 41.01% and 65.43% greater than that of CK under three drought stress treatments. The trend of the MDA content was consistent with that of the proline content. SOD activity was significantly increasing by 10.86%, 46.73% and 14.54% under three drought stress treatments. The activity of CAT in the SD treatment increased by 49.28%. All the indices recovered after rehydration. Furthermore, 54,78 and 51 different expressed metabolomics (DEMs) were identified in the LDCK/LD, MDCK/MD and SDCK/SD groups, respectively. There were 1,211, 1,265 and 1,288 different expressed genes (DEGs) were upregulated and 1,003, 1,819 and 1,747 DEGs were downregulated. Finally, combined transcriptomic and metabolomic analysis suggested that 437 DEGs and 24 DEMs of LDCK/LD group, 741 DEGs and 35 DEMs of MDCK/MD group, 633 DEGs and 23 DEMs of SDCK/SD group, were highly positively correlated in soybean plants under drought stress. Drought stress induced the expression of the *PAO1, PAO4, PAO5* and *P5CS* genes to promote the accumulation of spermidine and proline. Our study elucidates the responses of drought-stressed soybean plants in the field and provides a genetic basis for the breeding of drought-tolerant soybean plants.

## 1 Introduction

Drought, a prominent environmental factor, significantly reduces the growth and productivity of plants, thereby exerting adverse effects (Zhang et al., 2014; Tatar et al., 2020). Recently, the increasing warmth of the climate has notably intensified the severity of drought occurrences, rendering drought the most common natural disaster in terms of its impact on crop yields worldwide (Mahajan and Tuteja, 2005; Schmidhuber and Tubiello, 2007; Chen et al., 2013). Consequently, drought has emerged as a critical global concern (Daryanto et al., 2016; Wang et al., 2019a; Anwaar et al., 2020). Soybean (*Glycine max* L.), an important agricultural commodity, is widely cultivated and the largest plantation worldwide. As a plant with enough water to sustain growth, soybean yields are most affected by drought, with a 40% yield reduction (Silvente et al., 2012). Therefore, elucidating the drought-resistant mechanism of soybean plants is now a top priority, which will not only lay a theoretical foundation for improving the adaptability of soybean to drought stress, but will also promote the development of drought-resistant crop varieties and the improvement of crop yields.

When plants experience drought stress, they experience adverse effects such as morphological and structural changes, membrane damage, altered osmotic substances, inhibition of photosynthesis and stagnation of plant growth (Krasensky and Jonak, 2012). Leaves are the main functional organs involved in photosynthesis, and many studies have analyzed leaf photosynthesis (Bollig and Feller, 2014). Zivcak et al. (2014) revealed that drought stress negatively impacts both photosynthesis-related components and photosynthetic organs, while Singh and Raja (2011) indicates that plants exhibit varying responses to different levels of drought stress and employ diverse feedback mechanisms. The use of chlorophyll fluorescence enables precise and rapid assessment of plant health, offering insights into photosynthetic electron transport under drought conditions (Martinez-Ferri et al., 2015). Furthermore, plants can enhance their resistance to drought by modulating cellular physiology and biochemical metabolism, such as increasing the presence of cellular permeable substances. Furthermore, plants have the ability to withstand drought through modifications in cellular physiology and biochemical metabolism. This includes enhancing the presence of cellular permeable substances to uphold cell expansion pressure, augmenting cellular hydrophilicity and cell membrane permeability, activating endogenous protective mechanisms to boost the activity of antioxidant-related enzymes such as SOD/CAT, and nonenzymatic antioxidants, and regulating reactive oxygen species metabolism to counteract oxidative membrane harm (Ramirez-Villegas et al., 2018; Du et al., 2020; Wang et al., 2022a).

Enhanced plant resilience to drought stress can be attributed to alterations in morphological, cellular physiological, and biochemical characteristics, which in turn result in changes in metabolite and gene expression patterns. Prior research has shed light on the roles and molecular mechanisms of pivotal genes involved in drought stress responses, primarily by examining gene expression levels, molecular functions, and signal transduction pathways (Joshi et al., 2016). The advent of high-throughput sequencing technologies has introduced novel approaches, such as transcriptome analysis, that offer a robust means of investigating these relationships between molecular changes in plants. RNA-seq has been used to study the molecular mechanisms of drought stress resistance in soybean (Chen et al., 2016; Wang et al., 2022b). Analysis of the soybean transcriptome identified 213 drought-induced transcription factors, encompassing various families such as bHLH, ERF, MYB, NAC, and WRKY. Furthermore, a notable decrease in the expression of genes associated with chlorophyll synthesis and photosynthesis was observed, while an increase in the expression of genes linked to cell wall synthesis was noted (Chen et al., 2016). Utilizing RNA-seq, Song et al. (2016) conducted an investigation into the response of soybean to drought stress, revealing significant alterations in hormone signaling and metabolic pathways related to carbohydrates and cell walls. These findings imply that the identified genes may have pivotal roles in the plant’s response to drought conditions. KEGG analysis revealed that drought-stressed plants were involved in several molecular pathways, including ABA biogenesis, compatible compound accumulation, secondary metabolite synthesis, fatty acid desaturation, and plant transcription factor pathways (Xu et al., 2018). Therefore, exploring the response mechanisms of drought tolerance in soybean plants is essential for achieving high crop yields.

Several genes, signaling pathways, and metabolic processes participate in the plant response to drought stress. Metabolites are the direct expression and material basis of the physiological state of plants, and the accumulation of ABA and proline is the forms of plant response to drought stress. For instance, Li et al. (2022a) discovered a new mechanism governing the ability of wheat to withstand drought stress during the germination process utilizing a combination of metabolome and transcriptome analysis. Similarly, in response to drought conditions, plant secondary metabolite biosynthesis, amino acid metabolism, or amino acid synthesis pathways that reduce drought-induced effects were identified in chickpea plants (Khan et al., 2018). Furthermore, 236 differentially abundant metabolites involved in the proline biosynthesis, amino acid metabolism and biosynthesis of secondary metabolites contributed to the drought resistance of the leaves of *Jerusalem artichoke* seedlings (Zhao et al., 2021). Therefore, metabolomics, a highly sensitive and high-throughput method of metabolite identification, targets major metabolic pathways and identifies key regulatory factors, which can help to elucidate the mechanism of plant responses to stress.

In soybeans, drought stress occurs most commonly during the flowering stage and affects development and yield (Buezo et al., 2019; Yu et al., 2020). Therefore, to investigate the response mechanism and water-saving irrigation of soybean plants, ‘Liaodou 15’ cultivar plants were subjected to continuous with-held irrigation for 7 days (LD), 17 days (MD) and 27 days (SD) and then rehydrated until harvest. Physiological indicators, including antioxidant enzyme activities and photosynthetic parameters, were measured under drought stress and rehydration treatments. Then, transcriptomic and metabolomic analyses were performed to investigate several key genes and metabolic pathways in soybean plants under drought stress. Moreover, we analyzed the yield parameters of soybean plants in response to drought stress. The primary objective of this research was to elucidate the underlying mechanism governing soybean response to drought stress and to propose effective strategies for water-saving irrigation in soybean production.

## 2 Materials and methods

### 2.1 Plant materials and equipment

The test material used in this study was the drought-susceptible cultivar ‘Liaodou 15’, which about 500,000 acres of soybeans were planted in Liaoning province (Li et al., 2022b). The water control experiment was carried out with a sliding plastic film rain shelter at Shenyang Agricultural University (the scientific observation and experimental station for crop cultivation in Northeast China). The experiment was performed with brown soil. During the entire growth period, fertilizers were applied to 14-16-15 compound fertilizer (45%) and were used as basal doses of N, P2O5 and K2O, 52 and 56.25 kg·hm^−2^ were added to the soil before planting. Water irrigation was performed via a drip system.

### 2.2 Experimental design

The experiment adopted the field planting mode. This study used a random block design with three repetitions, and the size of each plot was 7.2 m^2^ (2 m × 3.6 m). The plants were planted in the north‒south monopoly direction, with a monopoly length of 2 m and a monopoly spacing of 0.6 m, of which the outermost two monopolies from the edge of the plot were 0.3 m, the plant spacing was 0.11 m, the plants were sown using hole sowing, and each plot had 108 seedlings. A total of 12 plots of three drought stress treatments were set up for soybean plants. Drought stress was induced at the early flowering stage (approximately 60 days after sowing) by continuously withholding irrigation for 7 days, 17 days and 27 days to reach mild drought stress (upper blade rolling, LD, 24.3% soil moisture content), moderate drought stress (leaf wilting and curling, MD, 20.6% soil moisture content) and severe drought stress (severe wilting and curling of leaves, SD, 16.9% soil moisture content). The control had a sufficient water supply (green and spreading leaves, LDCK, MDCK, SDCK, 30% soil moisture content). Moisture content was measured using SMTS-II-485 (China) sensor rods fitted with 30 cm sensors. After the completion of the drought stress period, the soybean leaves were sampled, and the plants in the rehydration treatment groups returned to control levels (30% soil moisture content) until harvest. Specific treatment methods were matched in a previous study (Li et al., 2022a).

### 2.3 Photosynthesis indices and chlorophyll fluorescence analyses

On the day of the end of the drought stress treatments and 5, 10 and 15 days after rehydration, we selected a sunny and cloudless day with good light and selected three plants with consistent and representative growth in each plot from 9:00 to 12:00; this process was repeated three times for each leaf, and inverted trilobal leaves of the soybean plant were selected and the light response curves were measured by using a portable photosynthesis tester (LI-6400XT, LI-Cor, Lincoln, NE, United States). The external CO_2_ concentration was maintained 400 μmol/mol, and the photosynthetically active radiation (PAR) was varied across 10 levels: 2,000, 1,600, 1,200, 900, 700, 400, 200, 100, 50, and 0 μmol/m^2^/s. The study quantified the photosynthesis rate (net photosynthetic rate, Pn), transpiration rate (Tr), and water use efficiency (WUE) of the plants. Additionally, chlorophyll fluorescence parameters were assessed in the central leaves of the inverted trifoliate leaves using a Fluor Cam fluorescence imaging system (FMS-2, Hansatech, UK) after a 20-minute dark adaptation period. The parameters measured included the maximum photochemical efficiency of PSII (Fv/Fm), non-optical quenching coefficient (NPQ), actual photochemical efficiency of PSII (ΦPSII), and photochemical quenching coefficient (qP).

### 2.4 Determination of proline and MDA contents and antioxidant enzyme activity

Samples were collected at 9:00 on the drought stress day and on the 5th, 10th, and 15th days after rehydration. Three plants were randomly selected from each replicate, with the sampling site being the top three leaves. The samples were frozen in liquid nitrogen and stored at −80°C. Leaf physiological indicators were quantified as follows: MDA and proline (Pro) content were measured using the thiobarbituric acid (TBA) reaction method. Additionally, catalase (CAT) activities were assessed using the iodometric titration method. Superoxide dismutase (SOD) activity was assessed using the nitrogen blue tetrazolium (NBT) photoreduction method.

### 2.5 Yield parameters

At harvest, 20 soybean plants were selected from each plot to measure relevant yield parameters, including plant height, main stem weight, pod number per plant, pod weight per plant, grain number, number of blighted soybean pods per plant, number of blighted grains per plant and 100-grain weight.

### 2.6 Soybean metabolomics analysis

Sample extraction and metabolite analysis were performed by Genedenovo Biotechnology Co., Ltd (Guangzhou, China). The metabolomics was analyzed with an LC‒ESI‒MS/MS system (HPLC, Shim-pack UFLC SHIMADZU CBM30A system; MS, Applied Biosystems 6500 Q TRAP) utilizing a Waters ACQUITY UPLC HSS T3 C18 column. The mass spectrometry conditions employed in this study involved the use of the API 6500 Q TRAP LC/MS/MS System, which was equipped with an ESI Turbo Ion-Spray interface and an electrospray ionization source (ESI) operating at a temperature of 500°C. The ion spray voltage (IS) was set to 5,500 V, while the curtain gas (CUR) was maintained at a pressure of 25 psi. Additionally, the collision-activated dissociation (CAD) parameters were set to high. Within the triple quadrupole (QQQ), each pair was scanned for detection based on the optimized declustering potential (DP) and collision energy (CE).

The filtering, peak detection and calculations of metabolomics data were performed using Analyst 1.6.1 software. Use variable importance (VIP) in projection to evaluate the relative importance of each metabolite in PLS-DA model. VIP ≥ 1, | log2 FC | ≥1 were considered a key identifier for metabolite differences (Takos et al., 2006).

### 2.7 Soybean transcriptomic analysis

Total RNA was extracted from drought-treated soybean leaves (NCBI SRA accession no. PRJNA852689). RNA integrity was evaluated using a Bioanalyzer 2,100 instrument (Agilent, United States). cDNA libraries were constructed and sequenced on the Illumina platform at Genedenovo Technologies Co., Ltd (Guangzhou, China). Clean reads were obtained and mapped to the soybean genome (https://phytozome-next.jgi.doe.gov/info/Gmax_Wm82_a4_v1) using HISAT2 software. DEGs were identified with *P* < 0.05 and fold change > 2 or fold change < 0.5 as the thresholds.

### 2.8 Statistical analysis

The data (mean values ± standard deviations) were analyzed by analysis of variance (ANOVA) using SPSS 20.0 and were analyzed with Microsoft Excel 2010. Duncan’s multiple range test at *P* < 0.05 indicated a significant difference.

## 3 Results

### 3.1 Phenotypes and yield parameters of soybean plants under different drought stress treatments

**FIGURE 1:**
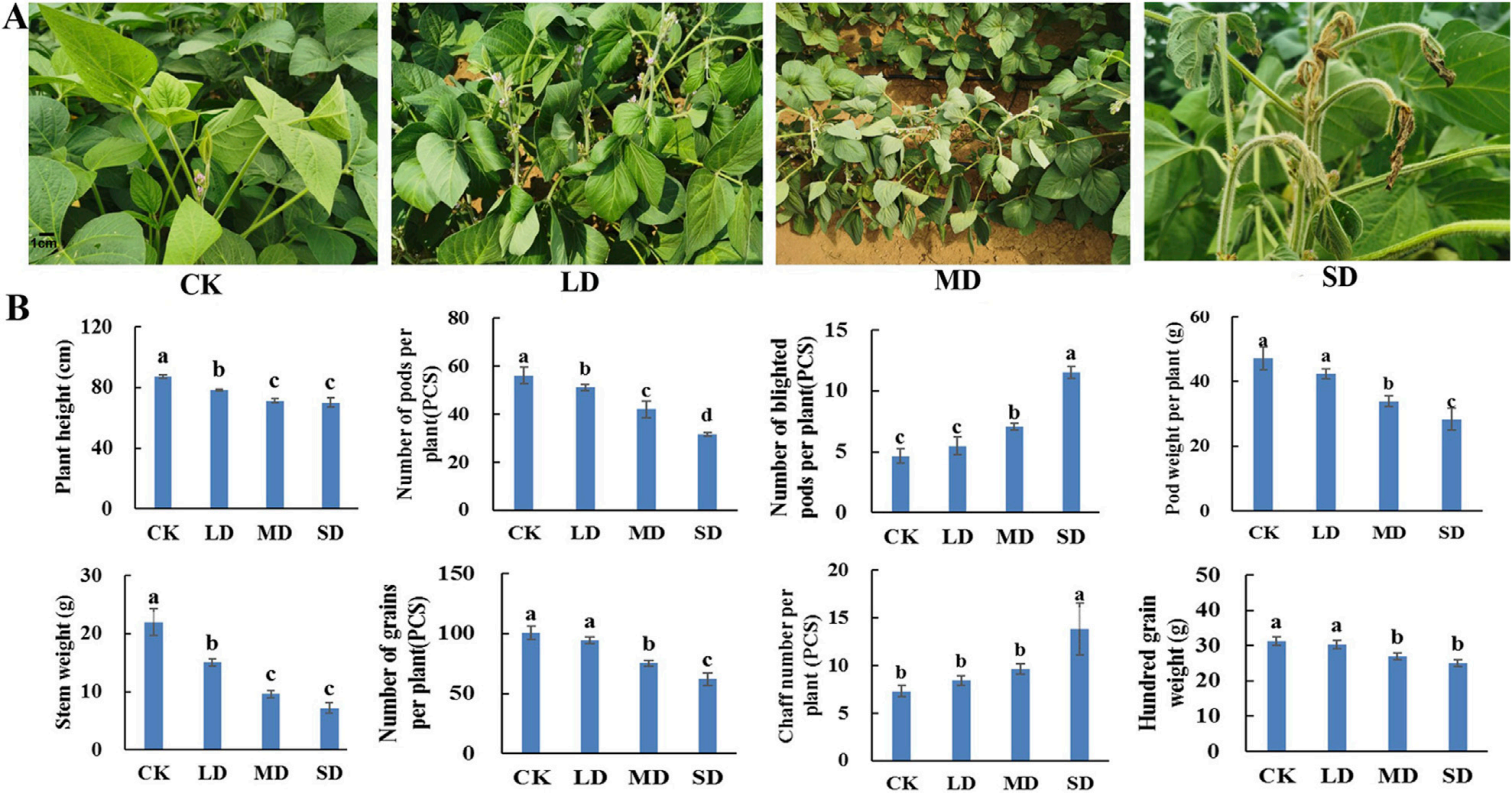
Determination of plant phenology and yield parameters under different drought stress treatments of soybean. **(A)** Phenotypes of soybean leaves. **(B)** The effect of different drought stress on soybean yield parameters.

In soybeans, drought stress occurs most commonly during the flowering stage and affects development and yield. The morphology of soybean plants, including leaf blade, plant height and stem weight, is affected by drought stress. With increasing water control time, the degree of drought stress in soybean plants increased, the upper leaves became yellow and curled, and the leaves gradually wilted and dried (Figure 1A). With increasing drought stress, the height of the soybean plants gradually decreased, and the height in the three drought treatments decreased by 11.8%, 22.4% and 29.0%, respectively, compared with that in the CK treatment. Similarly, the overall soybean stem weight was significantly lower than that in the CK group, with 22.4%, 38.2% and 51.7% decreases under the LD, MD and SD treatments, respectively (*P* < 0.05) (Figure 1B).

Furthermore, rehydration was carried out after drought stress, and a range of yield parameters were investigated. After drought stress treatment, the number of soybean pods per plant under the LD, MD and SD treatments decreased by 13.7%, 34.2% and 50.6%, respectively, compared with that under the CK treatment. With increasing drought stress, the number of blighted soybean pods per plant gradually increased, which was not significantly different from that of CK in the LD treatment and was significantly greater than that of CK in the MD and SD treatments by 61.2% and 135.6%, respectively (*P* < 0.05). The pod weights of the corresponding single plants under the MD and SD treatments decreased by 25.0% and 46.7%, respectively, which were significantly lower than those under the CK treatment (*P* < 0.05) (Figure 1B). Moreover, the number of soybean grains per plant decreased by 30.3% and 42.0% in the MD and SD treatment groups, respectively, which were significantly lower than that in the CK group, but the number in the LD group was only 4.2% lower than that in the CK group, and the difference between the LD and CK groups was not significant (*P* < 0.05). In contrast, the percentage of blighted soybean grains per plant in the three stress treatments was significantly greater than that in the CK treatment by 66.3%, 237.3% and 303.6%, respectively. The 100-grain weight of soybean plants under the MD and SD treatments differed from that under the CK treatment by 6.3% and 10.8%, respectively, while the 100-grain weight under the LD treatment was slightly greater than that under the CK treatment, which was more consistent with the pattern of change in soybean pod weight (*P* < 0.05) (Figure 1B). This indicates that soybean plants can withstand light drought without yield impacts but that both moderate and severe drought stress reduce soybean yields.

### 3.2 The photosynthetic rates and WUE of soybean plants under different drought stress conditions and rehydration treatments

**FIGURE 2:**
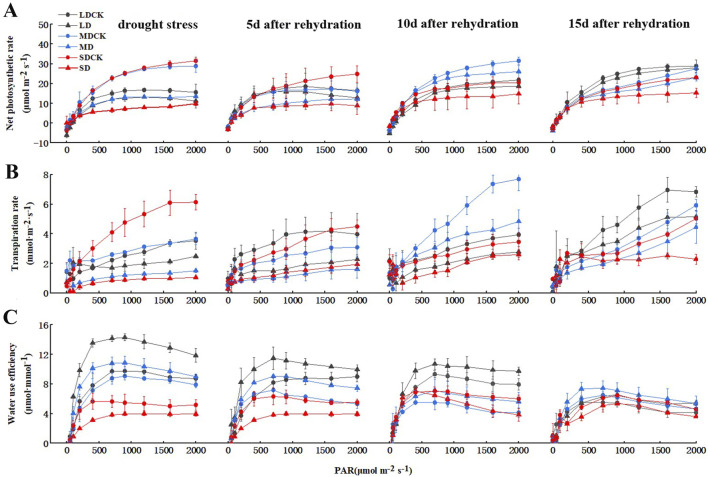
Changes of gas exchange parameters of soybean after drought stress. **(A)** Net photosynthetic rate. **(B)** Transpiration rate. **(C)** instantaneous WUE.

Drought stress is closely related to water use efficiency (WUE) and photosynthesis. To determine whether drought stress regulates photosynthesis, we generated photosynthesis–light curves for soybean plants under three drought stress treatments. The net photosynthetic rate of soybean decreased after different levels of drought stress. At a light intensity of 1,200 μmol m^−2^ s^−1^, net photosynthetic rates were 20.8% lower in LD than in LDCK, 51.5% lower in MD than in MDCK, and 71.8% lower in SD than in SDCK (Figure 2A). The net photosynthetic rate of the soybean plants gradually recovered over time after rehydration. The net photosynthetic rate of soybean plants after LD was similar to that after 15 days of rehydration. After MD and SD treatment, there was a certain degree of recovery over time after rehydration, and the net photosynthetic rate still did not recover to the level of that of MDCK and SDCK plants at 15 days but was still less than 15.7% and 25.5% of that of CK plants, respectively. This may have occurred because the soybean plants were damaged to a certain extent, and the damage was irreversible (Figure 2A). Leaf transpiration rate were markedly lower for SD treatments in comparison with SDCK. The transpiration rate of the soybean plants gradually recovered over time after rehydration (Figure 2B).

Furthermore, WUE increased rapidly in soybean plants at 700 μmol m^−2^ s^−1^ light intensity, followed by a small change with increasing PAR. As the degree of drought stress increased, the WUE increased by 31.8% under the LD treatment compared to that under the LDCK treatment and 31.5% under the MD treatment compared to that under the MDCK treatment, and the WUE under the SD treatment increased by 18.8% compared to that under the SDCK treatment. The plants gradually recovered after rehydration. Under the LD treatment, the overall WUE of soybean was greater than that under the other two treatments. With increasing rehydration time, the WUE gradually decreased from 28.6% greater than that in the LDCK treatment to 13.1% greater at the 700 μmol m^−2^ s^−1^ light intensity and slightly greater than that in the LDCK treatment at 15 days. The overall changes in WUE under the MD treatment were similar to those under the LD treatment, but the magnitude of the changes was slightly smaller. Under the SD treatment, the WUE was generally lower than that under the SDCK treatment at high light intensities, and although the WUE increased after rehydration, it was still 19.3% lower than that under the SDCK treatment at a light intensity of 700 μmol m^−2^ s^−1^ at 15 days after rehydration, probably due to the reduced degree of change in transpiration rate (Figure 2C). We speculate that soybean plant regulates drought stress to reduce the transpiration rate and increase the WUE and photosynthetic rate.

### 3.3 Chlorophyll fluorescence parameters of soybean plants under different drought stress conditions and rehydration conditions

When soybean plants were subjected to drought stress, the Fv/Fm and ΦPSII of leaf photosystem II decreased significantly (*P* < 0.05), and the Fv/Fm and ΦPSII of soybean leaf photosystem II decreased significantly by 14.3% and 10.6%, respectively, in the LD treatment group compared with in the LDCK treatment group, by 30.6% and 49.7%, respectively, in the MD treatment group, and by 63.9% and 54.7%, respectively, in the SD treatment group; additionally, the Fv/Fm and ΦPSII of photosystem II in soybean leaves in the SD treatment group were significantly lower than those in the SDCK treatment group (63.9% and 54.7%, respectively), and the differences among the stress treatments were significant (*P* < 0.05). Under the LD treatment, the values of both qP and NPQ were not significantly different from those under the LDCK treatment, differing by only 10.5% and 8.8%, respectively, whereas the values of qP and NPQ under the MD and SD treatments were significantly different from those under both the MDCK and SDCK treatments (*P* < 0.05), suggesting that there was little difference in the effect of appropriate levels of drought on the photosynthetic activity of photosystem II plants at the flowering stage (Table 1).

**TABLE 1:** Changes of chlorophyll fluorescence parameters of soybean after drought stress and rehydration treatments.

Treatments		Chlorophyll fluorescence parameters			
		Fv/Fm	ΦPSⅡ	qP	NPQ
Drought stress	LDCK	0.67 ± 0.05ab	0.64 ± 0.03a	0.50 ± 0.05a	1.10 ± 0.08ab
	LD	0.57 ± 0.04c	0.57 ± 0.02b	0.44 ± 0.04b	1.20 ± 0.03a
Rehydration	LDCK-5d	0.71 ± 0.09a	0.63 ± 0.01a	0.51 ± 0.03a	1.12 ± 0.04ab
	LD-5d	0.66 ± 0.02ab	0.59 ± 0.03ab	0.46 ± 0.03a	1.18 ± 0.07ab
	LDCK-10d	0.73 ± 0.02a	0.61 ± 0.02ab	0.49 ± 0.11a	1.08 ± 0.06c
	LD-10d	0.71 ± 0.07a	0.60 ± 0.04ab	0.48 ± 0.02a	1.10 ± 0.06bc
	LDCK-15d	0.72 ± 0.04a	0.60 ± 0.03ab	0.50 ± 0.01a	0.95 ± 0.03c
	LD-15d	0.74 ± 0.05a	0.64 ± 0.04a	0.49 ± 0.02a	0.97 ± 0.08c
Drought stress	MDCK	0.72 ± 0.04a	0.61 ± 0.02a	0.49 ± 0.11a	0.95 ± 0.03d
	MD	0.36 ± 0.03c	0.42 ± 0.02c	0.38 ± 0.01b	1.62 ± 0.01a
Rehydration	MDCK-5d	0.73 ± 0.06a	0.60 ± 0.01 ab	0.50 ± 0.06a	1.08 ± 0.06c
	MD-5d	0.44 ± 0.01c	0.45 ± 0.02c	0.43 ± 0.02b	1.34 ± 0.01b
	MDCK-10d	0.71 ± 0.09a	0.61 ± 0.06a	0.54 ± 0.10a	0.80 ± 0.05e
	MDCK-10d	0.59 ± 0.06b	0.54 ± 0.02b	0.49 ± 0.01a	1.04 ± 0.02c
	MDCK-15d	0.68 ± 0.03a	0.59 ± 0.07ab	0.51 ± 0.03a	0.93 ± 0.07d
	MD-15d	0.64 ± 0.06ab	0.61 ± 0.02a	0.51 ± 0.02a	0.95 ± 0.02d
Drought stress	SDCK	0.71 ± 0.09a	0.61 ± 0.02a	0.54 ± 0.10a	0.80 ± 0.07d
	SD	0.26 ± 0.06e	0.27 ± 0.03d	0.25 ± 0.02d	1.81 ± 0.06a
Rehydration	SDCK-5d	0.68 ± 0.03ab	0.59 ± 0.01a	0.51 ± 0.03a	0.93 ± 0.03c
	SD-5d	0.39 ± 0.04d	0.31 ± 0.01d	0.39 ± 0.02c	1.66 ± 0.04b
	SDCK-10d	0.61 ± 0.09ab	0.58 ± 0.05a	0.49 ± 0.01ab	0.80 ± 0.07d
	SD10d	0.46 ± 0.08cd	0.43 ± 0.01c	0.41 ± 0.01c	0.97 ± 0.05c
	SDCK-15d	0.56 ± 0.05bc	0.61 ± 0.02a	0.50 ± 0.03ab	0.72 ± 0.01d
	SD15d	0.49 ± 0.05cd	0.50 ± 0.03b	0.43 ± 0.02bc	0.93 ± 0.05c

Note: According to the LSD, the values of different lowercase letters differ significantly at the 0.05 level.

The values of all chlorophyll fluorescence parameters recovered after rehydration in soybean plants treated with LD and MD, with Fv/Fm, ΦPSII, and qP showing an increasing trend of recovery and NPQ showing a decreasing trend of recovery. Under LD treatment, there were no significant differences between Fv/Fm, ΦPSII, qP, or NPQ and LDCK at 5 days after rehydration, and the greatest degree of recovery occurred at 15 days after rehydration, with significant increases of 29 8%, 10 9%, and 11 4% for Fv/Fm, ΦPSII and qP, respectively, and NPQ significantly decreased by 19.2% (*P* < 0.05). Under MD treatment, the Fv/Fm, ΦPSII, qP, and NPQ significantly differed by 39.7%, 25.0%, 14.0% and 119.4%, respectively, from those of MDCK cells (*P* < 0.05). With the prolongation of time after rehydration, the expression gradually recovered to the level of that in MDCK cells at 15 days after rehydration. Under the SD treatment, the chlorophyll fluorescence parameters recovered to a certain extent with increasing time after rehydration, but ΦPSII was still significantly lower than that of the SDCK group by 18.0% at 15 days after rehydration, while NPQ was significantly greater than that of the SDCK group by 29.2% (*P* < 0.05) (Table 1).

### 3.4 The proline content, MDA content and antioxidant enzyme activity of soybean plants under different drought stress conditions and rehydration conditions

**FIGURE 3:**
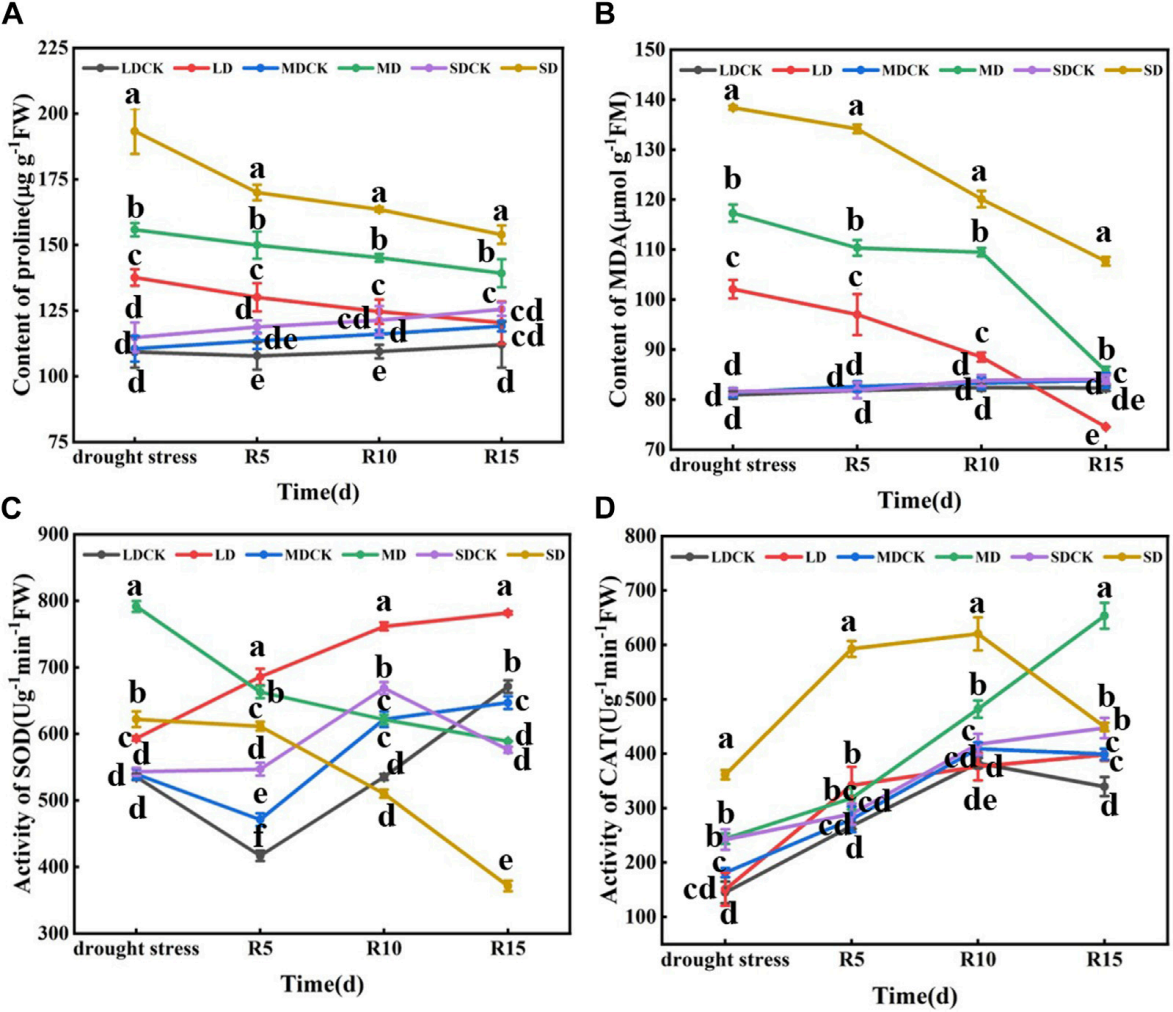
Determination of proline content, MDA content and antioxidant enzyme activity under different drought stress treatments of soybean. **(A)** Proline content. **(B)** MDA level **(C)** SOD activity. **(D)** CAT activity. The vertical bar indicates the standard deviation of three replications. For the same parameter, different letters denote a significant difference at *p* < 0.05.

Drought stress significantly affected the proline content of soybean leaves at the flowering stage, which was 25.94%, 41.01% and 65.43% greater than that of CK in the LD, MD and SD treatments, respectively, and 21.90% and 38.03% greater in the SD treatment than in the MD and LD treatments, respectively. The trend of the MDA content was consistent with that of the proline content, which was significantly greater by 26.13%, 44.04% and 69.57% in the LD, MD and SD treatments than in the CK treatment and by 14.90% and 35.58% in the MD and SD treatments, respectively, compared with the LD treatment. Drought stress during flowering significantly affected the SOD activity of soybean leaves, and SOD activity tended to increase and then decrease with increasing drought, significantly increasing by 10.86%, 46.73% and 14.54% in the LD, MD and SD treatments, respectively, compared with that in the CK treatment. Compared with that in the SDCK treatment, the activity of CAT in the SD treatment increased by 49.28%. The activity in the LD treatment differed by 14.90% and 35.58%, respectively, compared with that in the MD and SD treatments (Figure 3).

With the prolongation of rehydration, the proline content of soybean leaves after rehydration decreased but remained higher than that of the control. At 5–15 days after rehydration, the proline content was significantly greater in the SD group than in the LD and MD groups. Compared with those in the LD and MD treatments, the proline content in the SD treatment group was significantly greater than that in the other drought stress treatment group after rehydration. The proline content of the LD group was not significantly different from that of the control group at 15 days after rehydration. Similarly, there was a decreasing trend in the MDA content after rehydration following drought stress at the flowering stage. At 5 days after rehydration, significantly more plants in the different drought stress treatments than in the CK. At 15 days after rehydration, the MDA content in the MD and LD treatments returned to the control level, but the MDA content in the SD treatment was significantly greater than that in the other treatments and control groups. In addition, there was a decreasing trend in SOD activity after rehydration following drought stress at the flowering stage in soybean. At 5 days after rehydration, the SOD activity was significantly greater than that of the control under the different drought treatments, and at 10–15 days after rehydration, the SOD activity was lower than that of the control. In contrast, there was an increasing trend in rehydration CAT activity after drought stress at the flowering stage of soybean plants. From 5 to 15 days after rehydration, only the SD treatment resulted in greater CAT enzyme activity than did the other treatments and the control. Drought stress treatment at the flowering stage significantly increased the proline and MDA contents of soybean leaves and increased the CAT and SOD activities to counteract the damage caused by drought stress on the growth and development of soybean plants. After rehydration, the proline and MDA contents decreased and showed a negative correlation with increasing duration, showing a partial compensatory effect, and the compensatory effect became more obvious after rehydration following severe drought stress (Figure 3).

### 3.5 PAC, PLS-DA and differentially expressed metabolites (DEMs) of soybean under drought stress

**FIGURE 4:**
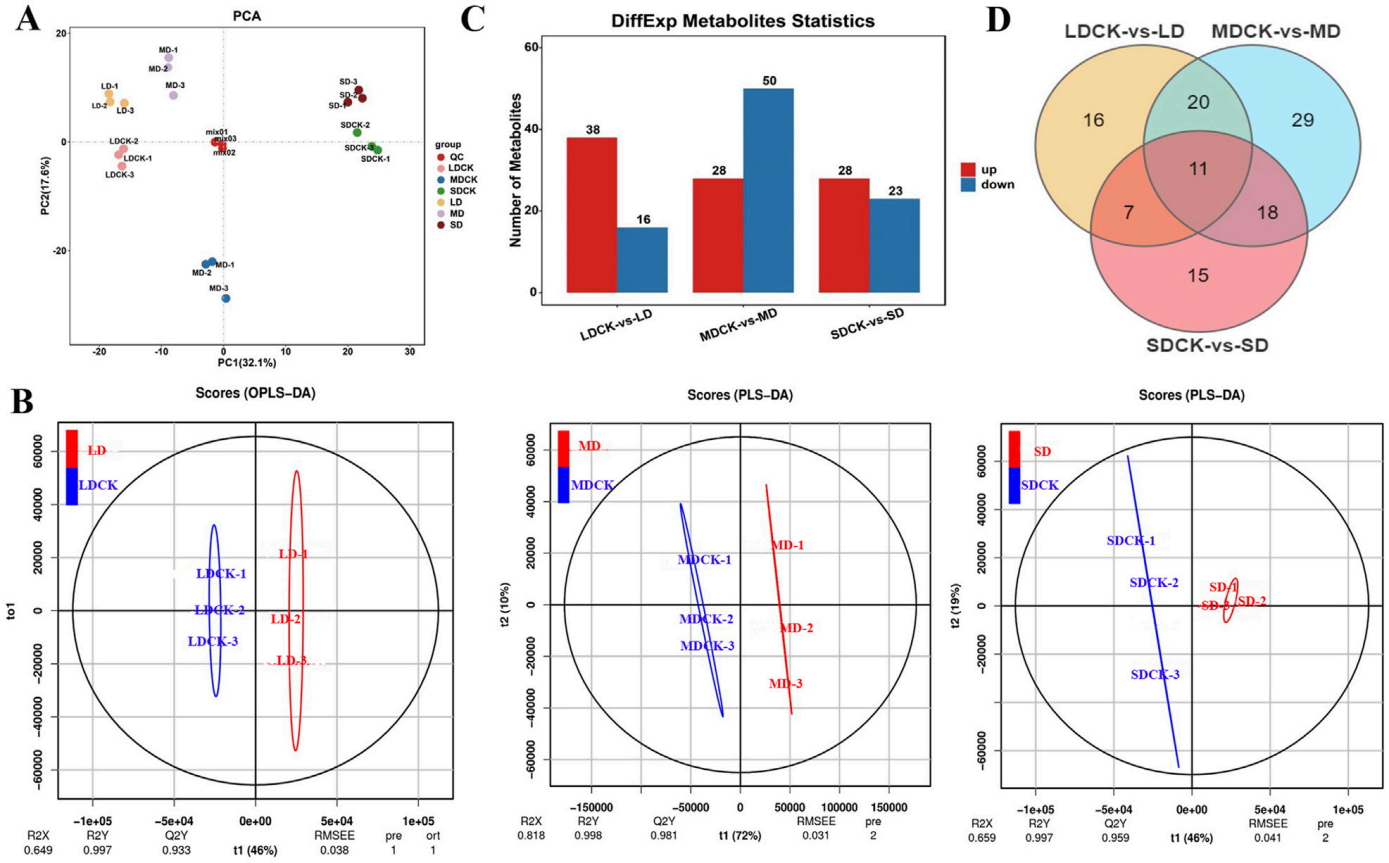
PCA analysis of soybean leaf samples after different drought stress treatments. **(A)** PCA analysis of soybean leaf samples after three drought stress treatments. **(B)** OPLS-DA score plots between the following pairs of groups: LDCK vs LD; MDCK vs MD; SDCK vs SD. **(C)** DEMs statistics in the leaves of soybean under drought stress. **(D)** Venn diagram for drought stress treatments.

Metabolomic analysis was performed on eighteen samples (three replicates of soybean after drought stress treatments and the corresponding controls) via principal component analysis (PCA), which revealed the overall intra- and intergroup metabolic differences among the LDCK, LD, MDCK, MD, SDCK and SD groups. As shown in Figure 4A, the samples within groups were well replicated with almost no differences, but the differences between the groups were significant, which indicates that drought stress significantly induced differences in metabolites. Moreover, paired analyses of the metabolic differences among the three different drought stress treatments are shown in Figure 4A. The first component (PC1) scores for LD, MD and SD were 40.6%, 56.4% and 43.9%, respectively, demonstrating that drought stress significantly affected soybean metabolism, especially under MD conditions (Supplementary Figure S1). The different metabolites of soybean plants under drought stress were identified by OPLS-DA. For LD, the R2X, R2Y, and Q2Y scores were 0.649, 0.997, and 0.933, respectively; for MD, the R2X, R2Y, and Q2Y scores were 0.818, 0.998, and 0.981, respectively; and for SD, the R2X, R2Y, and Q2 scores were 0.659, 0.997, and 0.959, respectively (Figure 4B). Additionally, cross-validation and permutation tests were completed on the OPLS-DA model to examine its reliability (Supplementary Figure S2). The model is stable and meaningful, indicating that drought stress affects metabolite levels.

Based on the FC values (|log2 FC| > 1) and PLS-DA model VIP values (VIP > 1, *P* < 0.05), DEMs were identified among the three drought stress treatments. A total of 54 DEMs were found in the LDCK/LD after drought stress, with 38 upregulated and 16 downregulated DEMs. There were 78 DEMs (28 upregulated and 50 downregulated) in MDCK/MD. In the SDCK/SD, a total of 51 DEMs were found, 28 of which were upregulated and 23 of which were downregulated (Figure 4C). To further understand the upregulated and downregulated variation in DEMs, differential fold changes were calculated for DEMs within the comparison group, and the DEMs volcano map was plotted based on the VIP value, FC value, and P value. A volcano plot was constructed to visualize the distribution of differentially abundant metabolites (Supplementary Figure S3). Furthermore, we identified 183 DEMs in LDCK/LD, MDCK/MD, and SDCK/SD, and 11 DEMs were commonly identified by Venn diagram (Figure 4D). A total of 183 DEMs were classified into 9 different categories, with the most variable categories being mainly alkaloids, flavonoids, lipids, amino acids and their derivatives. From Table 2, the various classes of metabolites can be seen in LDCK vs LD, MDCK vs MD, and SDCK vs SD.

**TABLE 2:** Number of DEMs in the leaves of soybean plants under drought stress.

Group class	LDCK-vs-LD		MDCK-vs-MD		SDCK-vs-SD	
	UP	Down	UP	Down	UP	Down
Alkaloids	15	1	8	8	8	4
Lignans and Coumarins	0	3	1	3	1	1
Phenolic acids	3	0	4	0	0	1
Flavonoids	6	1	7	15	6	1
Nucleotides and derivatives	0	4	1	3	0	2
Others	3	0	2	3	1	4
Lipids	4	6	0	8	2	9
Organic acids	2	0	2	1	2	1
Amino acids and derivatives	5	1	3	9	7	0
Total	54		78		51	

### 3.6 Functional annotation of DEMs and changes in the top 15 DEMs

**FIGURE 5:**
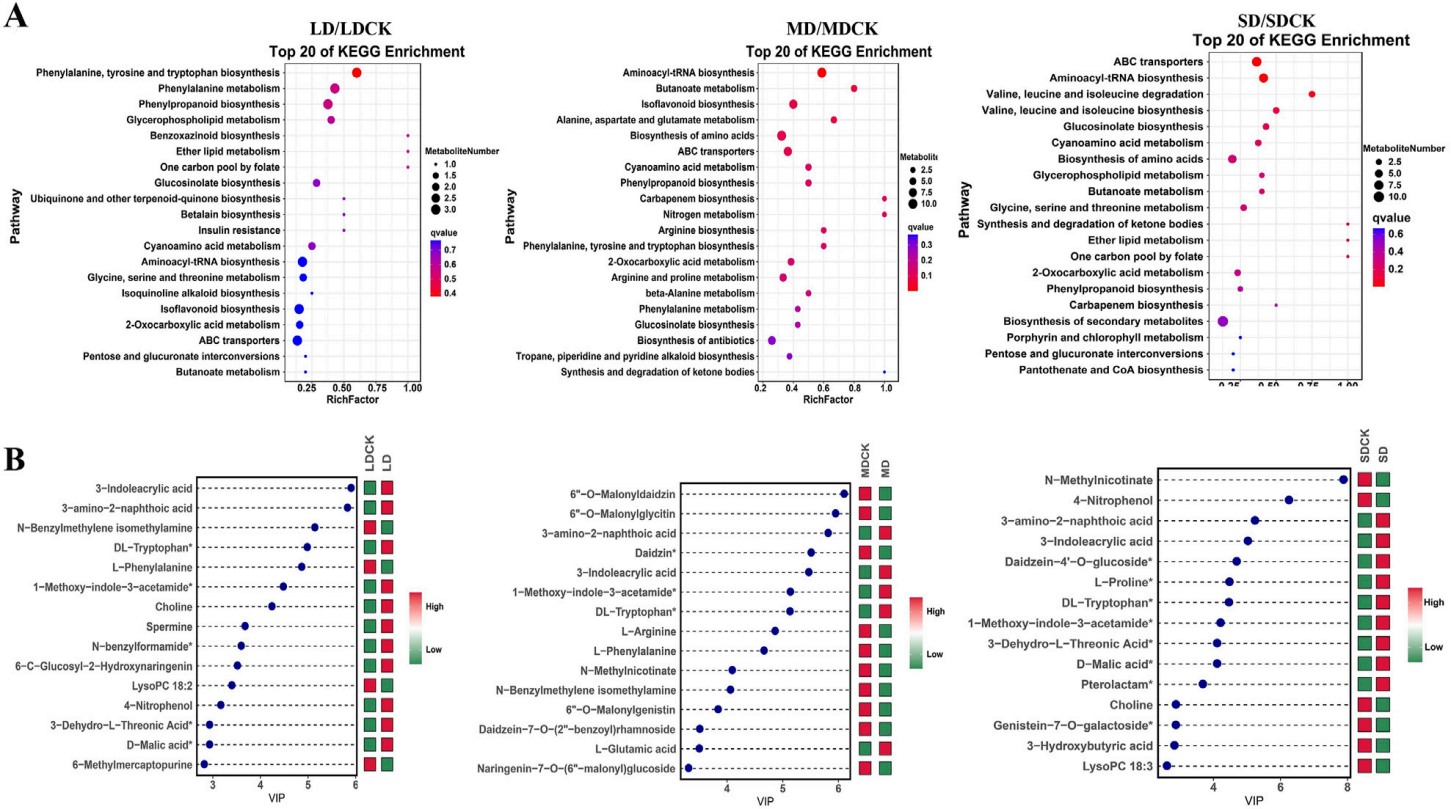
KEGG enrichment diagrams of DEMs and graph of log2FC values of the top 15 DEMs. **(A)** KEGG enrichment diagrams of DEMs in LDCK/LD, MDCK/MD, SDCK/SD. **(B)** Graph of log_2_FC values of the top 15 DEMs in LDCK/LD, MDCK/MD, SDCK/SD. Red indicates upregulation, green indicates downregulation. Abscissa shows log_2_FC and ordinate displays differential metabolites.

The KEGG pathway database is able to annotate and characterize differential substances enriched in different pathways. In the comparisons of the three groups of DEMs, a total of twenty pathways in which these metabolites participated in various stages were identified. Among these pathways, four are classified as biosynthetic pathways (aminoacyl-tRNA biosynthesis; indole alkaloid biosynthesis; glucosinolate biosynthesis and valine, leucine, and isoleucine biosynthesis), and the remaining are involved in primary metabolism. Aminoacyl-tRNA biosynthesis and phenylpropanoid biosynthesis were enriched under all three treatments. The phenylalanine, tyrosine and tryptophan biosynthesis and isoflavone pathways were enriched under LD and MD, and amino acid synthesis and phenylalanine metabolism were enriched under MD and SD (Figure 5A).

The changes in the top 15 DEMs among the three drought stress treatments can explain why soybean plants resist drought stress. Among the top 15 DEMs, 11 DEMs were high accumulated in the LD treatment (Figure 5B). In contrast, 5 DEMs were high accumulation under MD treatment. However, in the SD treatment, 5 DEMs were low accumulation (Figure 5B). Of these, 3-amino-2-naphthoic acid, 3-indoleacrylic acid, 1-methoxy-indole-3-acetamide and DL-tryptophan were high accumulated between soybean plants after the three drought stress treatments (Figure 5). In addition, among the low accumulation of DEMs, two DEMs (L-phenylalanine and N-benzylmethylene isomethylamine) were detected between the LD and MD treatments (Figure 5).

### 3.7 Analysis of the differentially expressed genes (DEGs) of soybean under drought stress

**FIGURE 6:**
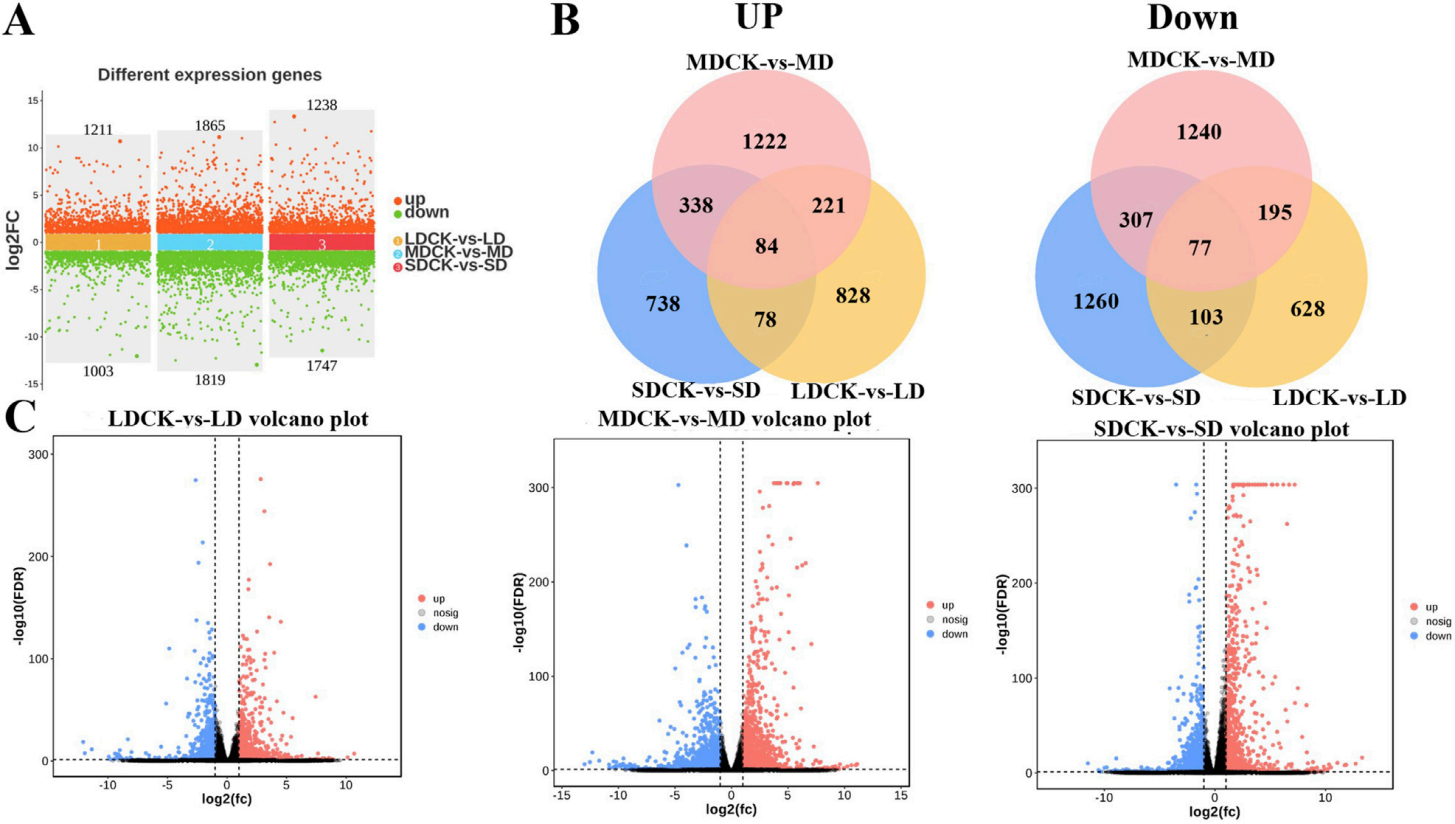
Analysis of transcriptome data in soybean after different drought stress treatments. **(A)** Numbers of DEGs in LDCK/LD, MDCK/MD and SDCK/SD. **(B)** Venn diagram of upregulated and downregulated DEGs in LDCK/LD, MDCK/MD and SDCK/SD. **(C)** Volcano plot of DEGs in LDCK/LD, MDCK/MD and SDCK/SD.

In order to further explore the regulatory responses of soybean plants to drought stress, the transcriptome of soybeans subjected to different levels of drought stress was determined. LD treatment had 2,214 DEGs of soybean compared to LDCK, with 1,211 genes upregulated and 1,003 genes being downregulated. A total of 3,684 DEGs was changed under MD treatment, of these, 1,865 were upregulated and 1,819 were downregulated. There were 2,985 DEGs that 1,238 genes being increased but 1,747 genes being down-expressed under SD treatment when compared to SDCK (Figure 6A). In addition, the expression of 84 genes being upregulated and the expression of 77 genes significantly repressed were synchronously expressed in both LD, MD and SD treatments (Figure 6B). Under different drought stress treatments, the change of DEGs in soybean leaves of MD treatment was greater than that of LD and SD treatment (Figure 6C).

**FIGURE 7:**
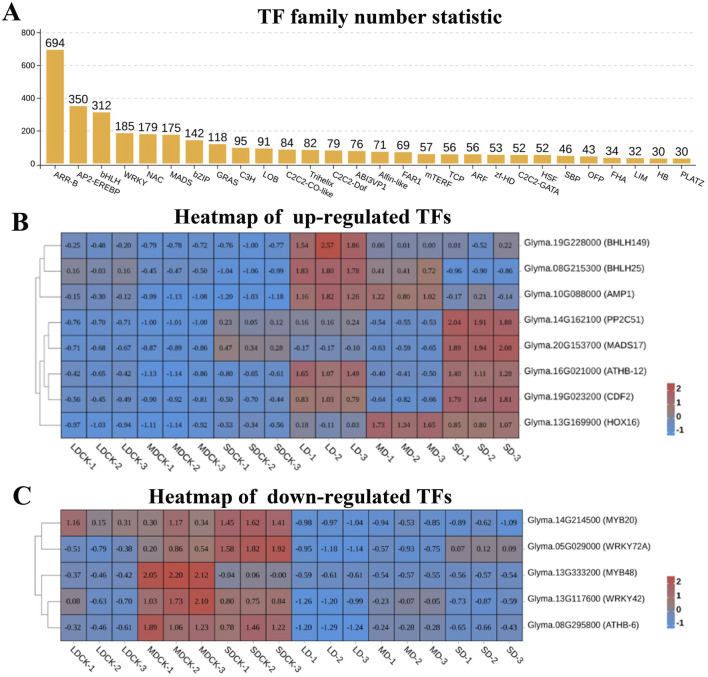
Analysis of transcription factors of soybean after different drought stress treatments. **(A)** Numbers of transcription factors family. **(B)** Heatmap of upregulated transcription factors. **(C)** Heatmap of downregulated transcription factors.

Transcription factors are major master regulators of drought stress response, among them, ARR-B is the transcription factor family with the highest number of DEGs, reaching 694 DEGs, followed by AP2/EREBP, bHLH, WRKY, NAC, MADS, bZIP, and GRAS transcription factor families, all of which have more than 100 DEGs (Figure 7A). According to the analysis of the Venn diagram, among the 84 upregulated genes, eight upregulated transcription factors were induced by three drought stress treatments. Among them, bHLH149 and bHLH25 were upregulated by higher multiples in LD and MD treatments, while MADS17 was significantly upregulated in SD treatment. It is speculated that they play a positive regulatory role in soybean resistance to drought stress (Figure 7B). Particularly, among the 77 downregulated genes, five downregulated transcription factors were induced by three types of drought stress, including two MYBs (MYB20 and MYB48) and two WRKYs (WRKY42 and WRKY72A), suggesting that they play a negative regulatory role in soybean resistance to drought stress (Figure 7C).

### 3.8 Joint analysis of the transcriptome and metabolome

**FIGURE 8:**
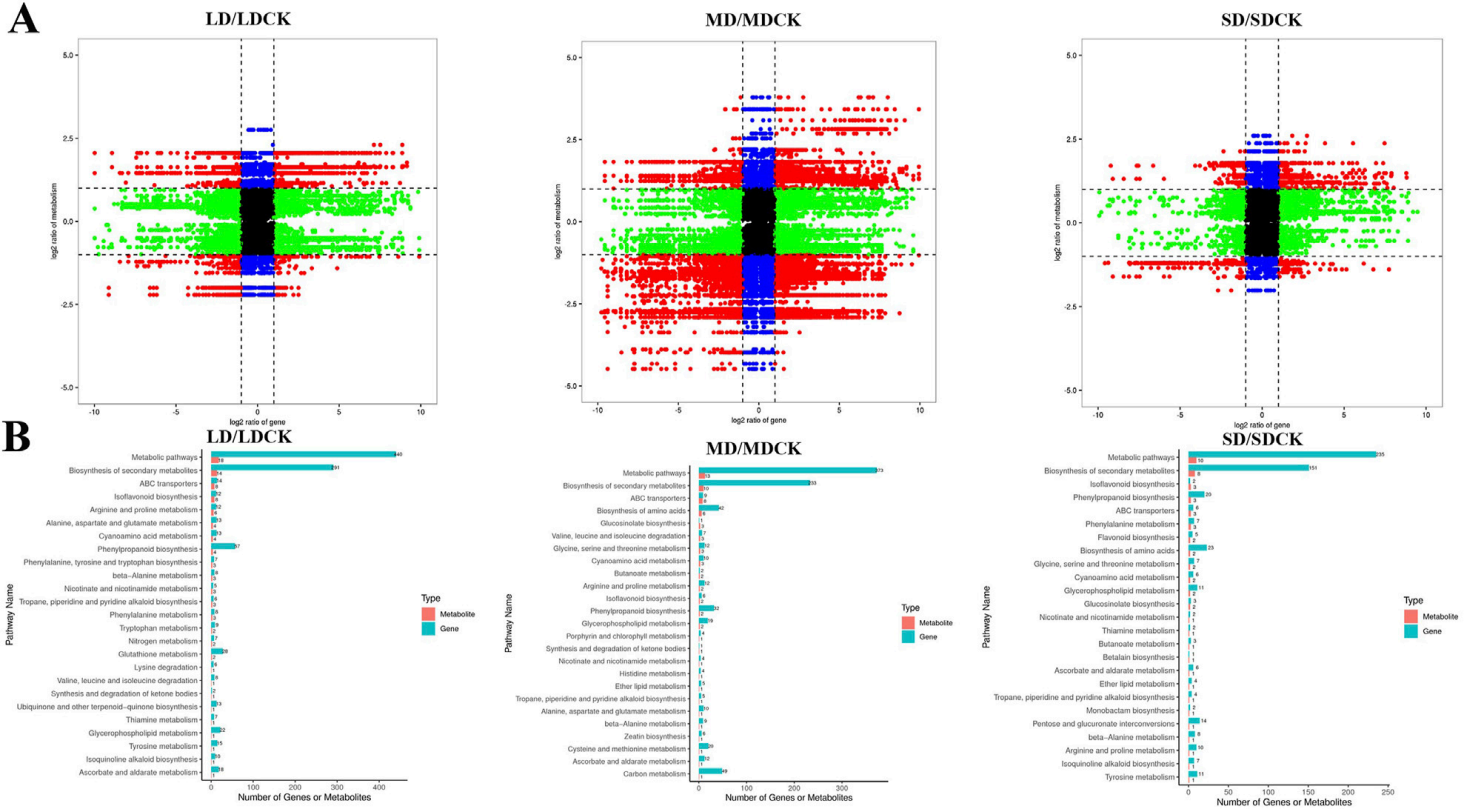
Correlation analysis of transcriptomic and metabolomic data from soybean plants at different drought stress treatments. **(A)** The nine-quadrant diagram shows the correlation between genes and compounds in three drought stress treatments. **(B)** KEGG enrichment analysis of DEGs (blue column) and DEMs (red column) enriched in the same pathway. Nine-quadrant plot: black points indicate non-differentially expressed metabolites and genes, red points indicate that genes and metabolites have the same or opposite trends, green points indicate that genes are differentially expressed but metabolites are not, and blue points indicate that genes are not differentially expressed but metabolites are differentially expressed. Cor absolute value > 0.99, *p* value < 0.5 and |log_2_FC| > 1.

To investigate the relationships between DEGs and DEMs in soybean leaves under drought treatments, a joint analysis of DEGs and DEMs was performed (cor absolute value >0.99 and *P* < 0.05). In the comparison of the three drought treatment groups, DEGs and DEMs were screened for mapping by |log_2_FC| > 1 only, and red dots indicate numerous genes were highly positively linked with metabolites (Figure 8A). These findings imply that related genes may either directly or indirectly control changes in the accumulation of these metabolites1 (Figure 8A). In LDCK/LD group, 437 DEGs and 24 DEMs was are highly positively correlated. In MDCK/MD group, 741 DEGs and 35 DEMs was are highly positively correlated. In SDCK/SD group, 633 DEGs and 23 DEMs was are highly positively correlated (Supplementary Tables S2–S4). According to the top 25 common pathways, KEGG enrichment analyses revealed that the DEGs and metabolites associated with the three drought treatments were enriched mainly in 7 biochemical pathways, such as “arginine and proline metabolism”, “ascorbate and aldarate metabolism” and “phenylpropanoid biosynthesis”, “Glycerophospholipid metabolism”, “cyanoamino acid metabolism,” “isoflavonoid biosynthesis” and “nicotinate and nicotinamide metabolism” (Figure 8B).

**FIGURE 9:**
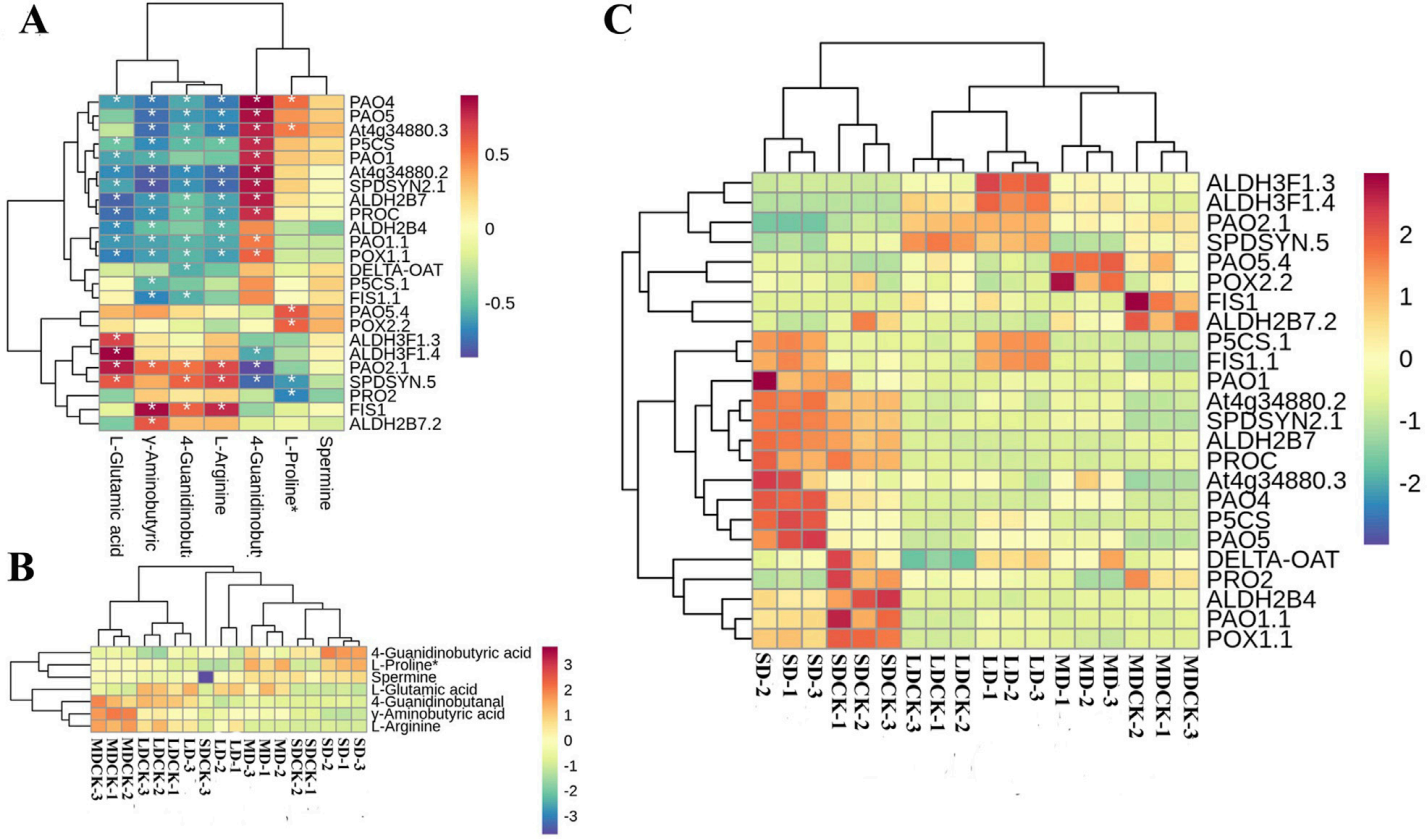
Arginine and proline metabolism pathways in soybean plants after drought stress of treatments. **(A)** Correlation heatmap of DEMs and DEGs in the biosynthesis of arginine and proline metabolism pathway under drought stress treatments. **(B)** DEMs are involved in arginine and proline metabolism pathway under drought stress treatments. **(C)** DEGs in the arginine and proline metabolism pathway under drought stress treatments.

According to KEGG enrichment, the “arginine and proline metabolism” was the most impacted biochemical pathway, 24 DEGs are correlated with seven DEMs (Figure 9A). Among the seven DEMs, L-proline and spermine, 4-Guanidinobutyric acid significantly accumulated in the three drought stress treatments compared to the CK, while the rest of the 4 DEMs decreased (Figure 9B). We further analyzed 24DEGs, the LD treatment significantly induced the expression of *ALDH3F1.3, ALDH3F1.4, P5CS.1* and FIS1.1. However, MD treatment induced an increase of *PAO5.4* and *POX2.2* genes expression. It is worth noting that SD treatment significantly upregulated 7 DEGs, including *PAO1*, *PAO4*, *PAO5* and two *P5CS* genes related to proline and spermine content. This implies that the activation of the arginine and proline metabolism pathway in response to LD drought treatment, as opposed to control treatment, may primarily facilitate plant growth, while SD conditions may enhance resistance to osmotic stress through the accumulation of proline in soybean plants (Figure 9C).

## 4 Discussion

Drought, an abiotic stress, limits crop growth and development (Caser et al., 2018). Under drought stress, plant growth patterns and adaptation mechanisms produce changes in plant physiology and biochemistry, genes and metabolites, suggesting that some metabolic pathways and genes play important roles in enhancing drought resistance (Silvente et al., 2012; Ashraf et al., 2018). The present study was carried out by controlling water, observing plant growth status and determining physiological indicators (photosynthesis characteristics, chlorophyll fluorescence parameters, and WUE) and yield parameters after drought stress and rehydration treatments, while the response of soybean plants in the field to drought via continuous water control was investigated via transcriptomics and metabolomics.

The reduction in crop yield and quality caused by drought is mainly attributed to the fact that drought affects plant growth and development through significant physiological, biochemical and molecular changes (Bhargava and Sawant, 2013). The findings of this study align with previous research showing that the soil water content continued to decrease as the duration of water control increased (Abbas et al., 2018). Under different degrees of drought stress, plants exhibited leaf curling, plant dwarfing and reduced soybean yields (Figure 1A). After rehydration, soybean plants exposed to LD and MD conditions exhibited an enhanced capacity for recovery, potentially through a reduction in aboveground biomass and leaf area as part of their drought tolerance mechanism. In contrast, SD soybean plants exhibited a decrease in 100-grain weight and pod number and did not show satisfactory recovery after rehydration, likely because drought stress inhibited soybean plant growth (Figure 1B). The plant yield after rehydration depends on whether key physiological processes and functions are disrupted (Abid et al., 2018). In our previous study, the physiological changes and differentially expressed genes of soybean plants under drought stress were studied (Li et al., 2022b). With the extension of the water control time, there was a significant increase in the MDA and proline levels, as well as the CAT activity, following the SD treatment. SOD activity increased under LD and MD stress, indicating that osmo-regulatory substances as well as antioxidant enzyme activity are important for maintaining soybean plant growth after plants are subjected to different levels of drought stress (Figure 3). This conclusion is consistent with previous studies (Das et al., 2015).

Drought is one of the most important extreme weather and climate events (AghaKouchak et al., 2021). The rate of photosynthesis, which refers to the rate at which photosynthesis fixes carbon dioxide (or produces oxygen), is an important factor affecting plant growth and has a relatively sensitive response to drought events (Massacci et al., 2008). In the present study, the rate of photosynthesis of soybean plants gradually decreased with increasing intensity of drought stress at the flowering stage, with an increasing difference compared with that of the CK plants (Figure 2A), which may be attributed to the fact that the plant xylem or the roots of the plant sense insufficient water when the soil water supply is reduced, which can lead to the closure of the stomata and a decrease in photosynthesis and water loss (Kimm et al., 2020). At 5 days after rehydration, some photosynthetic characteristics were partially restored, but there was still a gap between them and the CK group, and they were basically restored to the CK level at 10 and 15 days after rehydration; moreover, the rate of photosynthesis in the SD group did not recover to the CK level at 15 days after rehydration, which was more consistent with the findings of previous studies (Qi et al., 2021). In addition, the plants in the LD treatment group had significantly greater photosynthesis rate than did those in the LDCK group at 15 days after rehydration, which appeared to indicate a supercompensation phenomenon, suggesting that moderate drought may improve the drought tolerance of plants (Figures 2A, B).

Furthermore, water use efficiency (WUE) is a comprehensive indicator for evaluating plant growth suitability under water deficit conditions, and under future climate change, WUE will continue to change as the frequency and severity of drought increase (Liu et al., 2015). Enhancing water use efficiency (WUE) serves as a crucial mechanism for crops to attain drought tolerance, conserve water, and achieve high yields. Various studies have demonstrated that the impact on crop water consumption escalates notably with heightened drought severity and prolonged stress duration. In this study, we found that the WUE under drought stress was generally greater than that under CK, and the WUE of soybean increased significantly at 5 days after rehydration in the LD and MD treatments and was close to that under CK at 10–15 days after rehydration (Figure 2C), which may be because after rehydration under MD stress, the plant self-regulated by reducing transpiration, and the plant itself did not suffer from serious damage; moreover, when the soil moisture was replenished in time, the use of deep water in the soil was promoted by soybean. Promoted deep soil water utilization by soybean. WUE still decreased after rehydration under SD stress and was slightly lower than that under CK, suggesting that at this time, the transpiration rate decreased less, stomatal dominance decreased, and nonstomatal limitation became the dominant factor in the decrease in the photosynthetic rate.

The absorption and conversion of light energy by plants are mainly divided into three closely related components: chlorophyll fluorescence, photosynthetic electron transport, and heat consumption-related components (Wang et al., 2018). It is generally believed that drought stress leads to changes in photosynthetic properties, which are mainly reflected in the injury to the PS II active center, resulting in the blockage of its electron transport function, the occurrence of photoinhibition, and the reduction of the efficiency of the primary light reaction, which in turn causes changes in chlorophyll fluorescence parameters (Wang et al., 2018). Some studies have shown that under drought stress, a decrease in the rate of electron capture by photosystem II leads to a decrease in photochemical efficiency and a different degree of recovery after rehydration (Qi et al., 2021). In this study, Fv/Fm, ΦPSII, and qP continued to decrease with increasing drought stress intensity during the flowering stage of soybean plants and were significantly lower than those of the CK plants, indicating that a certain intensity of drought caused an obvious photoinhibition phenomenon. All of the plants recovered to varying degrees after rehydration and recovered to exceed the level of the CK 15 days after rehydration under MD stress, which was more in line with the changes in photosynthesis rate (Table 1), suggesting that moderate drought stimulated the protection of soybean plants against stress and that the photochemical efficiency of light system II decreased. In this study, soybean mainly consumed excess light energy by increasing heat dissipation to reduce the effects of drought, and self-regulation under drought conditions in different plants and varieties needs to be further explored.

Metabolic profiling analysis has been utilized to elucidate the intricate metabolic processes associated with stress response and regulation. Numerous metabolomic investigations have been carried out on various plant species under conditions of drought-induced stress (Hong et al., 2020; Wan et al., 2021). For instance, in tea plants, a total of 166, 401, and 334 metabolites exhibiting differential accumulation were identified in the CK vs MI, CK vs MO, and CK vs SE comparison groups, respectively. These metabolites included a wide array of amino acids and their derivatives, organic acids, nucleotides and their derivatives, isoflavones, and glycosyl flavonoids, all of which were found to be responsive to drought stress (Li et al., 2020). Various metabolites accumulate in different plant species in response to various drought stress factors. For instance, *Pisum sativum* leaves exhibit a significant increase in amino acid contents such as proline, valine, threonine, homoserine, inositol, r-aminobutyric acid, and trigonelline (Adrian et al., 2008). *Capsicum annuum* leaves primarily accumulate fructose, sucrose, galactinol, cadaverine, putrescine, and spermidine (Astrid et al., 2010). *Trifolium pratense* predominantly accumulates rosinol, proline, and malic acid (Steven et al., 2014). In our research, 54, 78, and 51 DEMs were identified using LC-MS in the LDCK/LD, MDCK/MD and SDCK/SD comparison groups, respectively (Figure 4C). Among them, DL-tryptophan and L-glutamic acid were significantly accumulated under MD and SD treatments (Figure 5B). This indicates that these amino acids play important roles in drought tolerance in soybean. This discovery aligns with previous research findings. Jia et al. (2016) observed 16 amino acid alterations that were notably prevalent under moderate drought conditions but significantly reduced under severe drought conditions. Rabara et al. (2017) reported that amino acids accounted for 33% of the differentially abundant metabolites observed in tobacco and soybean plants under drought stress. These findings collectively demonstrate that amino acids and their derivatives play a pivotal role in plant responses to drought-induced stress. At the same time, proline, an osmoregulatory substance, significantly accumulated only in soybean plants under LD and MD stress (Figure 9). Notably, proline has been identified as a potential enhancer of cell tolerance and protector against damage caused by diverse abiotic stresses (Zhu et al., 1998; Ashraf and Foolad, 2007). The proline levels of barley, wheat, and sunflowers increased, indicating that these plants are involved in mitigating the effects of drought stress. Chmielewska et al. (2016) analyzed metabolite changes in barley under drought stress and observed the accumulation of proline in the leaves of both tested barley cultivars. Similarly, Wang et al. (2019b) reported a significant accumulation of proline in drought-tolerant wild soybean plants. Hence, the significant presence of proline in plants under drought conditions suggests that proline plays a crucial role in the response to drought stress.

The integration of transcriptomic and metabolomic analyses provides valuable insights into the intricate mechanisms by which plants respond to drought stress at a holistic level (Arun et al., 2014). Prior investigations have delved into the molecular underpinnings of plant resilience to drought, pinpointing specific genes such as *HY5, GST, NCED* and *P5CS*, as well as crucial metabolites like phenylalanine, proline, and flavonoids. Additionally, a range of metabolites associated with phenylpropanoid biosynthesis, starch and sucrose metabolism, and proline have been identified, shedding light on essential biosynthetic and secondary metabolic pathways (Savoi et al., 2016; Dalal et al., 2018; You et al., 2019; Zhao et al., 2019; Min et al., 2020). In the present study, we have successfully identified key genes (*P5CS, PAOs*) and three metabolites (spermidine, proline and phenylalanine) involved in amino acid biosynthesis and metabolism (Figures 9A–C).

## 5 Conclusion

In this study, we investigated the regulatory responses of soybean plants at the blooming stage under various drought stress conditions simulated using water control methods in field crops. Physiological, photosynthetic, and chlorophyll fluorescence parameters and yield indicators in soybean plants under drought stress and rehydration treatments were measured. The results suggested that the resistance of soybean plants to drought stress increased by altering the photosynthetic rate and chlorophyll fluorescence parameters; increasing the MDA content, proline content and SOD activity; and decreasing the CAT activity. Moreover, varying levels of drought stress had a significant impact on the weight of 100 grains and the number of pods, resulting in an increased rate of abortion. Furthermore, metabolome analyses were carried out and suggested that soybean plants can activate isoflavone, amino acid biosynthesis, and phenylalanine metabolism pathways in response to drought stress, which leads to the accumulation of four amino acid metabolites and secondary metabolites. Finally, combined metabolome and transcriptome analysis revealed that drought stress enhances resistance by upregulating the *P5CS* and *PAO* genes to increase the proline and spermidine contents. Overall, we have improved the understanding of the molecular mechanism by which drought stress affects soybean plants, which can serve as a valuable resource for breeding soybean plants with enhanced resistance to drought stress.

## Data Availability

The datasets presented in this study can be found in online repositories. The names of the repository/repositories and accession number(s) can be found below: https://www.ncbi.nlm.nih.gov/, PRJNA852689.
