# Supplementary figures and images for: Metabolomics combined with transcriptomics and physiology reveals the regulatory responses of soybean plants to drought stress
Source: Front Genet. 2024 Oct 24;15:1458656. doi: 10.3389/fgene.2024.1458656 (PMC11541050; doi:10.3389/fgene.2024.1458656)

| **pair** | **pre** | **R2X(cum)** | **R2Y(cum)** | **Q2(cum)** |
| --- | --- | --- | --- | --- |
|  |  |  |  |  |
| LDCK-vs-LD | 1+1 | 0.649 | 0.997 | 0.933 |
| MDCK-vs-MD | 1+1 | 0.818 | 0.998 | 0.973 |
| SDCK-vs-SD | 1+1 | 0.659 | 0.997 | 0.938 |

**Table S1.**

**
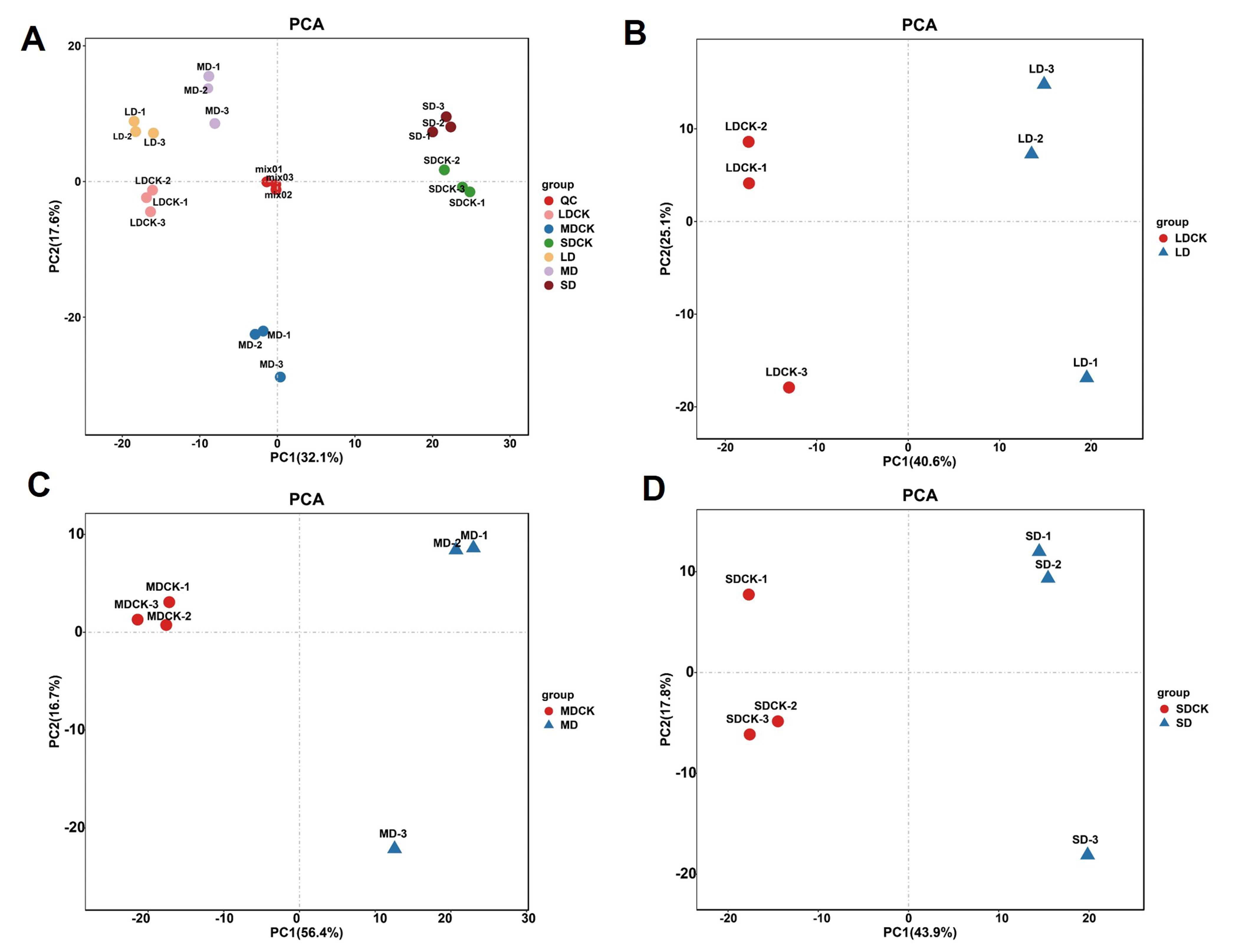
**

**Figure S1**


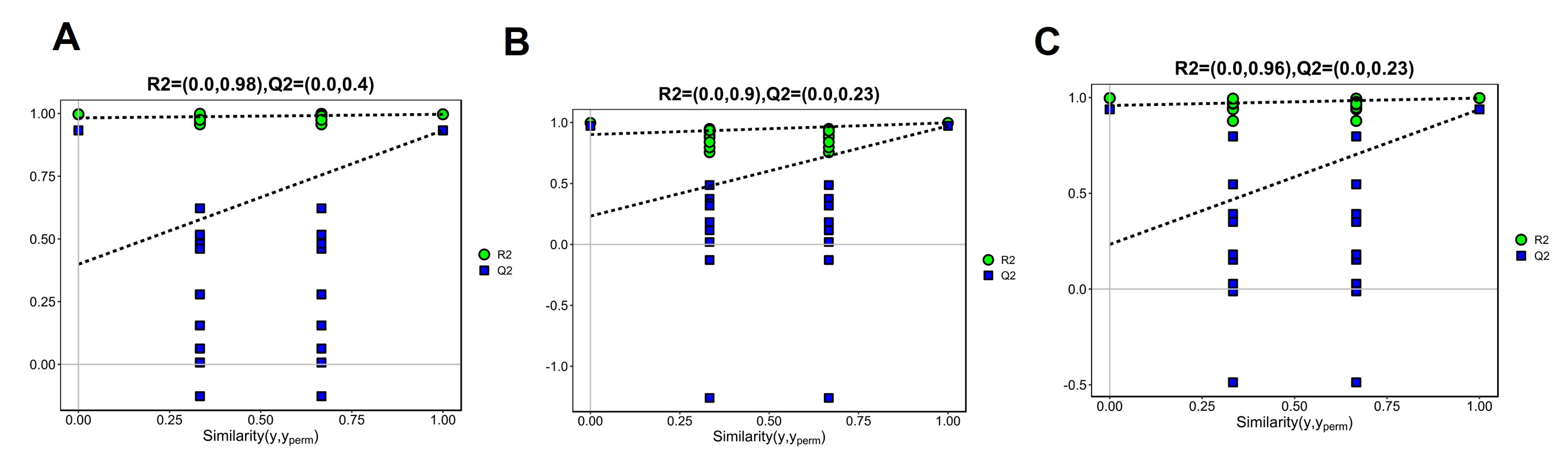


**Figure S2**

**Figure S3**

**
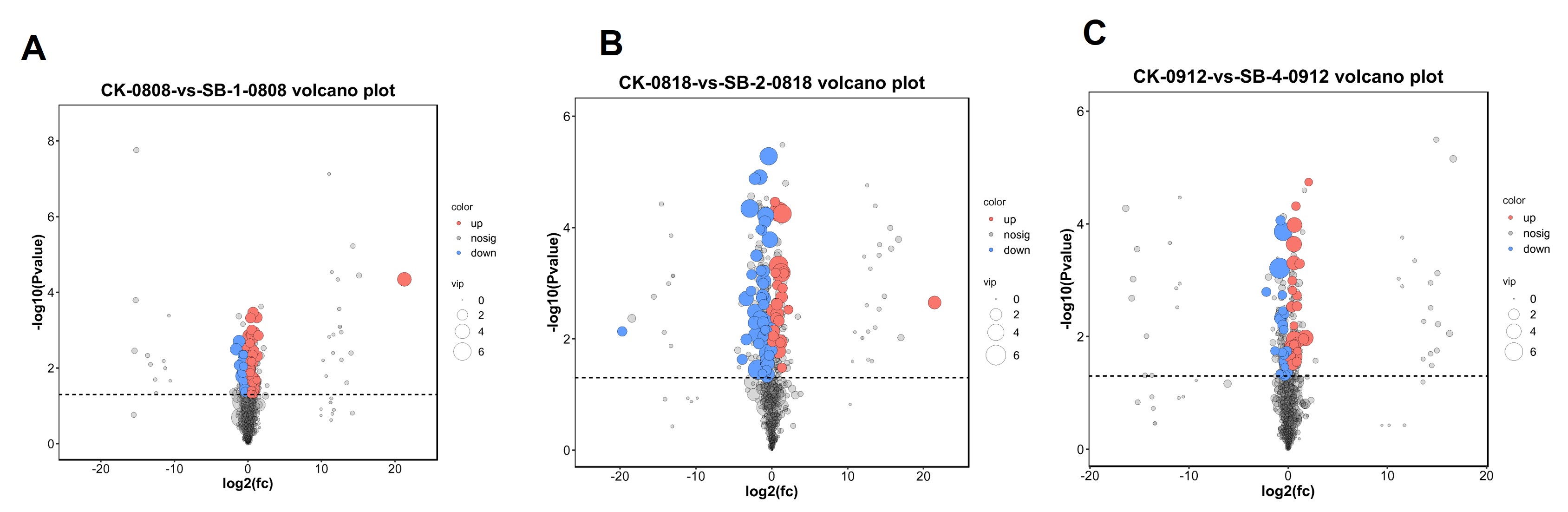
**

Supplement: Supplementary file 3 [file Table1.docx]
